# ADPO: Adaptive DRAM Controller for Performance Optimization

**DOI:** 10.3390/mi16040409

**Published:** 2025-03-30

**Authors:** Zhuorui Liu, Yan Li, Xiaoyang Zeng

**Affiliations:** 1 School of the Academy for Engineering and Technology, Fudan University, Shanghai 200433, China; 19110860029@fudan.edu.cn; 2 The State Key Laboratory of Integrated Chips and Systems, School of Microelectronics, Fudan University, Shanghai 200433, China; xyzeng@fudan.edu.cn

**Keywords:** memory systems, data movement, main memory, energy efficiency, memory scaling, low-latency computing, DRAM controller, bank parallelism, page open policy, DRAM page policy

## Abstract

Emerging applications like deep neural networks require high off-chip memory bandwidth and low dynamic loaded Double Data Rate SDRAM (DDR) latency. However, under the stringent physical constraints of chip packages and system boards, it is extremely expensive to further increase the bandwidth and reduce the dynamic loaded latency of off-chip memory in terms of DDR devices. To address the latency issues in DDR subsystems, this paper presents a novel architecture aiming at achieving latency optimization through a use case sensitive controller. We propose a reevaluation of conventional decoupling mechanisms and quasi-static arbitration methods in the DDR scheduling architecture. The adaptive scheduling algorithms offer significant advantages in various real-world scenarios. The research methodology involves implementing a rank-level timing aware read/write turnaround arbiter and setting read/write queue thresholds and read/write turnaround settings based on observed patterns. By implementing the arbiter and dynamically adjusting these parameters, the proposed architecture aims to optimize the performance of the DDR subsystem. To validate the effectiveness of the architecture, we conduct multiple experiments. These experiments evaluate the performance of the DDR subsystem under various workloads and configurations. The results demonstrate that the adaptive scheduling algorithms have advantages in achieving DDR performance attributes for workloads and improving system performance. The experimental results provide evidence of the architecture’s effectiveness in reducing latency by around 10% to 50% in various real-world scenarios.

## 1. Introduction

The rise of big data and deep learning has brought about revolutionary breakthroughs in artificial intelligence in application fields such as vision, speech, and language. The data scale and algorithm scale of upper-layer applications are progressively increasing day by day. This leads to the cache being unable to cover the memory footprint as effectively as before, and the demand for hardware computing capability is growing exponentially. As Moore’s law starts to slow down and Makimoto’s wave still exists, the fabrication enhancements at the silicon level can no longer provide the predictable and extensive gains in computer performance.

The high latency of off-chip memory accesses has long constituted a critical bottleneck to thread performance. This issue is further exacerbated in chip multiprocessors where memory is shared among concurrently executing threads. The bandwidth and latency of a memory system are significantly influenced by the interaction between memory access and the “3D” structure of banks, rows, and columns inherent to modern DRAM chips. Therefore, we hold that it is of utmost importance to focus on hardware architecture, algorithm development, and software performance engineering to continuously improve computer applications in this new era [1,2].

To deal with this DDR performance issue, previous research efforts have focused on utilizing the upmost DDR technologies, like near memory computing, to offload identical workload near DRAM. This includes PIM [3] and AiM [4]. Despite this progress, the high costs in DRAM manufacturing and the extensive modifications needed in both hardware and software have restricted their wide adoption. Prior works [5,6] have relied on DRAM internal buffers to eliminate these read and write turnarounds, which have to modify the DRAM internal structure. This makes the solution relatively expensive, and then it is not possible to directly use the widely used DRAM devices. We extend the scope of previous work by solving this problems in a DDR controller scheduler, which is a much more easily adopted solution.

In summary, our work represents a novel approach to optimizing system power consumption by adapting the performance configurations of the memory controller based on traced data. By extending the scope of previous work to include emerging memory technologies, we aim to contribute to the development of more energy-efficient computing systems.

This work makes the following contributions:

1. Different from traditional DRAM controllers, we have employed a decoupled architecture of scheduler and protocol, which enables a scene-based adaptive DRAM controller with dynamic adjustments.
2. By considering the read–write mode switching penality, we have designed a dynamic read and write mode arbiter to improve DDR utilization.
3. In terms of dynamic threshold control strategies, we have also compared the performance benefits brought by this technology. It leads to average read latency savings ranging from approximately 10 to 150 ns.

## 2. DRAM Adapitive Control Arbiter

### 2.1. Theoretical Basis

In order to effectively optimize the overall performance, we target the fundamental issue of memory bandwidth utilization by addressing pattern impact and scheduling methods. Read/Write Switching: As the DRAM timing speed continues to increase, the read/write turnaround constrained by DRAM timings such as tWTR and tRTW is also on the rise. At a latency value of approximately 40 DRAM TCK cycles, it becomes equivalent to the page hit/miss turnaround latency.

As shown in Table 1, both RTW (Read to Write) and WTR (Write to Read) have a very significant penalty, which will lead to a degradation of DRAM performance. Furthermore, the read and write turnaround has the greatest delay penalty. However, for different ranks, this switching has almost no penalty. The timing for read to write switching within the same rank has almost the same penalty as that in different ranks. For different ranks, the timing between read to read or write to write is almost double that within the same ranks. The gap from write to read within the same rank is the largest, and we should try our best to avoid this transition.

**Figure 1:**
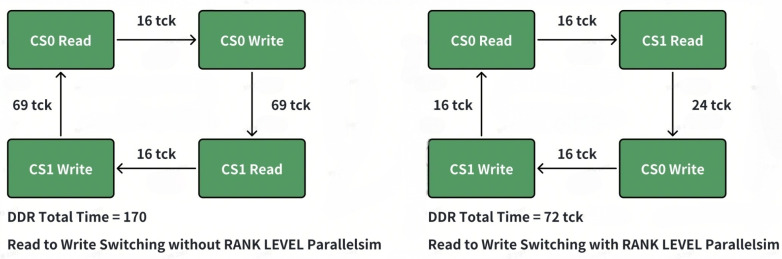
Read to write switching penality difference.

From Figure 1, we can observe that the penalty difference shows a total increase of more than 136%. Implementing a proper read and write switching policy that takes into account timeout, address collision, and read and write queue levels is of utmost importance.

### 2.2. Read and Write Switching Engine

To address the read and write switching penalty, we employed the read–write mode arbiter in both the Address Queue and Bank Queue, taking into account rank-level parallelism.

**Figure 2:**
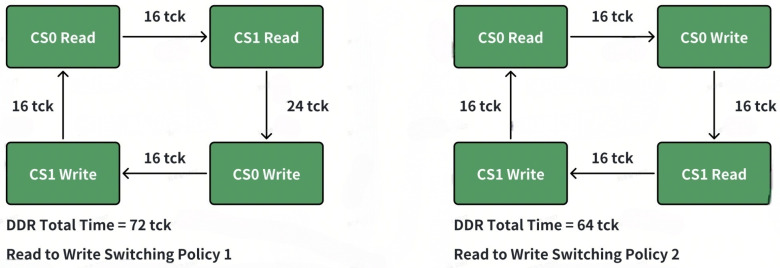
DDR mode switching policy options difference.

**Figure 3:**
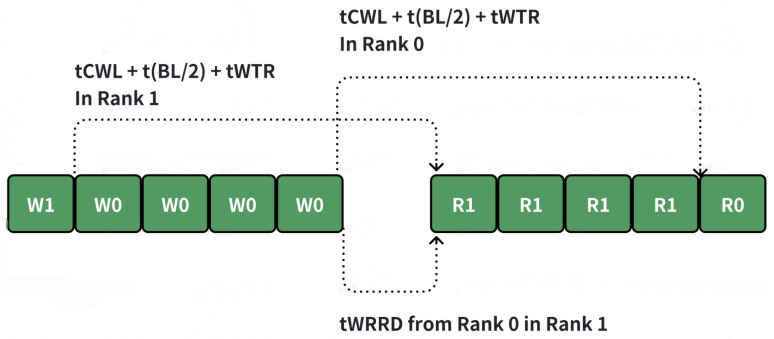
Switching policy option 2 benefit.

For a two-rank system, there are four directions. As mentioned above, we must ensure that the write-to-read switching is a rank-level switch. There are two options below to achieve this. As illustrated in Figure 2 and Figure 3, option 2 will create fewer bubbles and achieve higher utilization.

Compared with option 1, the write to read path is much longer, which will also increase the possibility with the same rank write to read switch. For example, suppose Scheme 1 is adopted, the current mode is CS0_WR, and the next mode is CS1_WR. However, if the number of commands in CS1_WR is small, it will switch to CS0_RD. When the number of CS1_WR is less than tccd_wtr_spr/tccd_s, additional bubbles will appear (as shown in the figure below). But if Scheme 2 is adopted, as long as there is a sufficient number in CS1_RD, tccd_wtr_spr will not occur.

### 2.3. Adaptive Scheduling Engine

**Figure 4:**
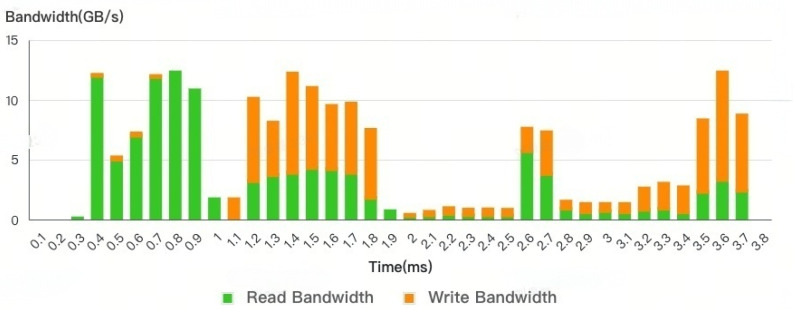
DDR bandwidth requirement variations across timeline.

Furthermore, as depicted in Figure 4, the traffic in any use cases will result in the bandwidth requirement for read and write being dynamically changed. In contrast to existing DDR controllers with static or quasi-static software-controlled registers, the current approach overcomes the limitations of scheduling methodology by enabling a dynamic tuning of performance settings. This enables the DDR controller to effectively adapt to trace requirements within short time intervals, such as on the order of microseconds.

We achieve this adaptive scheduling methodology at the controller structure level, unlike the DDR controller in previous solutions [7,8,9,10,11,12,13] which is designed with a single thread, causing the DDR read/write command to be judged only in a single arbiter engine. Consequently, the active/precharge command is generated only with the read/write command, leading to inefficient DDR utilization. Each active/precharge command in parallel banks has to wait to be scheduled after the bank is closed. However, there is another improvement in DRAMSim3 [14], where the active/precharge command can be arbitrated in a separate channel and can be arbitrated when the bank read/write command takes the local control complex timing parameters. Our approach is based on multiple threads. As each transaction generates both read/write and active/precharge commands, the active/precharge commands are arbitrated concurrently. Thus, this is a true multiple thread where the read/write command is transformed into the precharge/active command, and the precharge/active command is arbitrated with DDR utilization first in parallel with read/write command arbitration. The arbiter is within the local control timing. Therefore, arbitration and local timing control are separated.

Moreover, the up-to-date configuration can be accessed through this method. After investigating the impact of scheduling conflicts on DRAM performance, the results show that contention for bandwidth among different masters can lead to scheduling conflicts, thereby resulting in a decrease in system performance. Furthermore, the row buffer (internal cache line) of DRAM has a significant impact on the turnaround time for both page hits and misses. The read/write turnaround time is constrained by DRAM timing parameters such as tWTR and tRTW. The interference generated by DRAM refresh operations affects consecutive transactions similar to PPT training and ZQ calibration. Conflicts can also occur when processing transactions that are smaller than one burst and not address aligned, necessitating the splitting or merging of transactions.

From the perspective of the key component level of the controller, since it enables data transactions between different masters and DRAM, the controller is mainly composed of three blocks: AQ (Address Queue), BQ (Bank Queue), and PROTOCOL.

AQ (Address Queue) is designed to receive AXI (Advanced eXtensible Interface) requests and reorder them according to specific rules. This reordering enables downstream components to receive the requests in a more efficient way, ultimately enhancing SDRAM access.

BQ (Bank Queue) is designed in a Content-Addressable Memory (CAM) style. Transactions are stored in this CAM with the objective of balancing delay and bandwidth. The grant of active and read/write commands takes into account various factors, including QoS (Quality of Service), store status, bank and page hit information, and more. The BQ also features pre-charge/active and refresh interfaces to establish communication with the Protocol block.

PROTOCOL is primarily responsible for implementing the requirements defined in the DDR specification to ensure the proper operation of DRAM. It receives transaction commands from the BQ and issues the corresponding commands to the DDR PHY. The Function part of the protocol is dedicated to maintaining the correct working status of DRAM and implementing the low-power features of DRAM, thereby optimizing power consumption.

**Figure 5:**
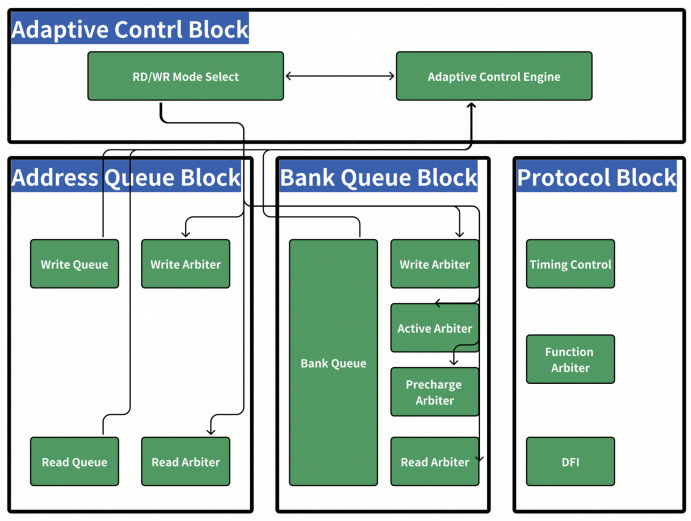
Adapitive control diagram.

As it is extremely difficult to handle more than 350 DRAM timing parameters simultaneously, the scheduler in the DDR controller previously mainly dealt with page hit and page miss conditions. We utilized the capability of making pipeline adjustments in response to changes in bandwidth and other relevant characteristics, as shown in Figure 5.

The adaptive control engine retrieves the address queue and bank queue internal attributes to calculate a shifting result for the better handling of performance tuning. Currently, the adaptive scheduler focuses on addressing increased delays caused by GPU case frame buffer flushing. Consequently, the implemented pipeline adjustment algorithm is relatively simple and straightforward. However, as a future step, there is a plan to introduce a gradual adjustment mechanism and utilize optimization algorithms to determine the optimal hyperparameters. The current adjustment mechanism operates in two aspects: one is in read and write turnaround settings, and the other relies on water level tuning. Meanwhile, we apply the QoS methodology to attempt to eliminate this issue by allocating bypass entries for high QoS transactions. This approach aims to reprioritize foreground applications to prevent them from experiencing starvation. Since foreground applications typically consume less bandwidth compared to high-load computing use cases, the impact of high QoS should be manageable. (1) Regarding the unfairness among different transaction types with different workloads, we agree that unfair resource allocation can degrade performance across diverse workloads. However, in this controller for read and write transactions, for write transactions, the response is sent back immediately after the write data burst is completely received in the write data RAM. Thus, from the perspective of this write-transaction-type unfairness, at the system level, the reduced read latency is more beneficial to SoC performance. (2) Regarding the unfairness among the same transaction type with different workloads, the main goal of this work is to improve the overall performance, especially the average read latency. The unfairness resulting from this should be relatively small and can be addressed by the QoS and virtual channel features presented in this paper. Moreover, it can be orthogonal to other works that enhance fairness.

## 3. DRAM Adapitive Control Arbiter Implemetation

**Figure 6:**
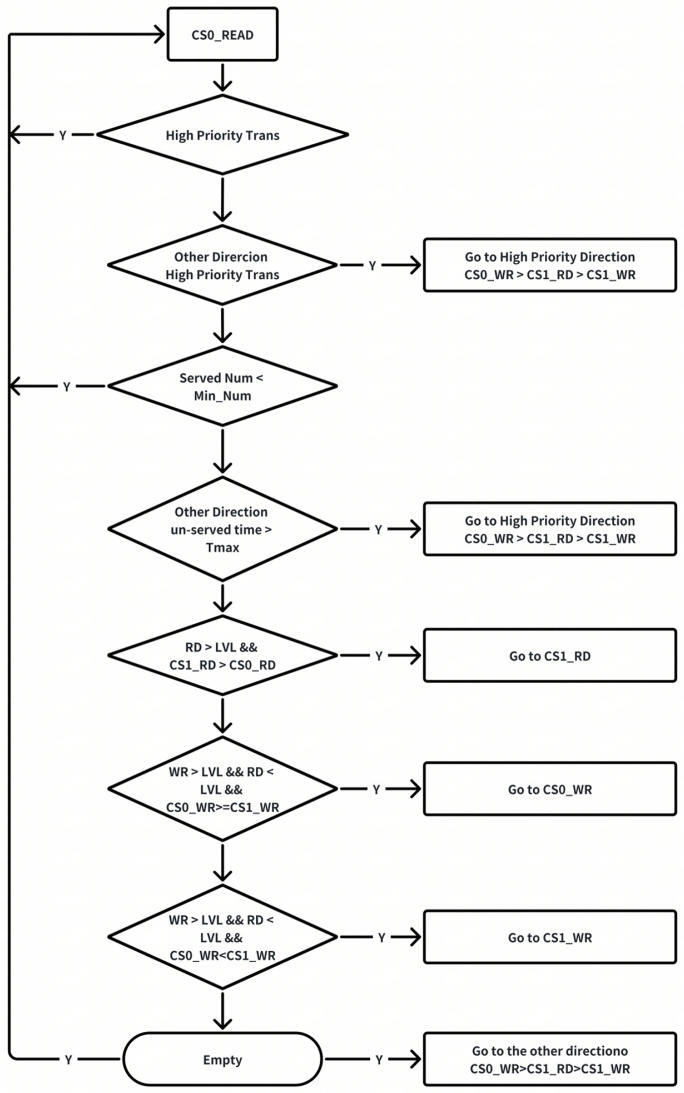
Read and write switching flow.

The intention of the read–write mode arbiter is to ensure that switching does not occur too frequently. When switching occurs, it must follow the diagram below in Figure 6. The read/write swiching methodology is generally based on the following considerations.

(1) If the read–write switching is accomplished well enough, theoretically, the number of transactions in the four modes should be balanced. However, due to the following reasons, certain modes may accumulate a relatively large number of commands.
(2) There is a certain imbalance in the access to different ranks, and there is also an imbalance in the ratio of reading to writing. This may be because the upstream hash or interleave is not good enough, or the access pattern during a certain period has spatial locality. For the overall read–write switching, whether it is ACT or RW, it is more inclined toward reading. Write commands are relatively more difficult to arbitrate, which may lead to a backlog of write commands. In such a situation, it is necessary to switch to the corresponding mode to eliminate this imbalance.
(3) The timing parameters are unbalanced. For example, the time from WR to RD (TCCD) within the same rank is significantly longer than the other timing parameters. This naturally makes it difficult for the write operations within the rank to switch to read operations. Thus, proper rank-level parallelism is used to reduce the bubble when read and write turnarounds have to be initiated.

Regarding dynamic control, we take the GFX benchmark scenarios as an example, and the following conditions need to be met: the Master Write Large Indication and Write Level are both at Level 2, the BQ Level is Level 2, and there is a decrease in Page Hit Count accompanied by an increase in Page Conflict Count and Miss Count. Then, the Read/Write Timeout Value and BQ Level 1 Value will be increased by adding an offset. However, if the Master input decreases and the internal level no longer satisfies the defined conditions, the offset will be removed.

Currently, the purpose of the adaptive scheduler is to accommodate the increased delays resulting from GPU case frame buffer flush. As such, the implemented algorithm for pipeline adjustments is relatively simple and straightforward. However, future plans include implementing a more gradual adjustment mechanism and utilizing optimization algorithms to determine the hyperparameters. The current adjustment mechanism consists of two stages.

**Figure 7:**
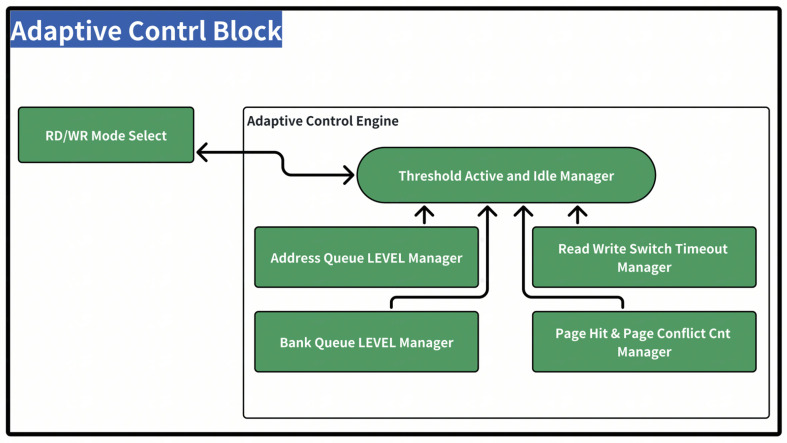
Adaptive control module diagram.

As depicted in Figure 7, the following principles regulate the behavior of the system: (1) If the depth of the AXI Write Queue (AWQ) is greater than or equal to the AW level queue 1 (aw lvlq1), and the difference between the Bank Queue level queue 1 (bqlvl1) and the depth of the Bank Queue (BQ) is greater than a configurable parameter (currently set at 1), then the BQ waterline 1 will be increased to a configurable value (currently set at 30). When the condition is not satisfied, it will revert to the default value of 26. (2) If the depth of the AWQ is greater than or equal to the AWQ level queue 1 (awqlvl1), and the ratio of AW bytes to AR bytes (aw byte/ar byte) is greater than a configurable parameter (currently set at 2), the timeout value for read/write mode switching will be reduced to a configurable value (currently set at 120). Otherwise, it will return to the default value of 256.

## 4. Results

### 4.1. Experimental Methodology

To achieve this goal, a cycle-accurate simulator based on SystemC was constructed during the evaluation and integrated into the Electronic System Level (ESL) platform to form a comprehensive simulation environment. By modeling the behaviors of the Address Queue (AQ), Command Queue (BQ), protocol engine, DDR PHY (Physical Layer), and DRAM device, the performance and efficiency of the proposed design can be accurately evaluated. We consider a dual-rank LPDDR5-8533M with a total of 32 banks and a 64-bit data wide data bus. Throughout the evaluation process, by analyzing the simulation results, we can deeply understand the impact of the adaptive adjustment of the read/write switching impact on the entire system, thereby verifying the effectiveness of our method and making adjustments or optimizations as needed.

Table 2 summarizes the key parameters used in our experiments.

As for the workload, when evaluating our adaptive control module, in the case of GPU-only chips, the DRAM density requirement is relatively low. Single-rank DRAM mitigates the PISI (Power-Induced Signal Integrity) impact associated with multi-rank DRAM, which could otherwise degrade the DDR (Double Data Rate) performance. However, in multi-core and CPU/GPU co-existing systems, a high DRAM density is crucial. In particular, with the emergence of LLM scenarios, the memory footprint has become larger. To meet this high density requirement, a multi-rank DRAM configuration is typically employed. Compared to the CPU which is a latency-sensitive master, as shown in Figure 4, the GPU is a heavy-loading master, especially the GPU-related benchmars, and the GFX benchmark is the memory bound workloads. Therefore, we used the GPU benchmark platform to validate our concepts. To obtain the request traces, we execute GPU benchmarks in an architecture simulatior. During the simulation, trace players read the requests from the traces and generate the appropriate commands. After comparing each frame of the whole GPU benchmark, the heavy loading frame F-181 was selected.

### 4.2. Performance Results

**Figure 8:**
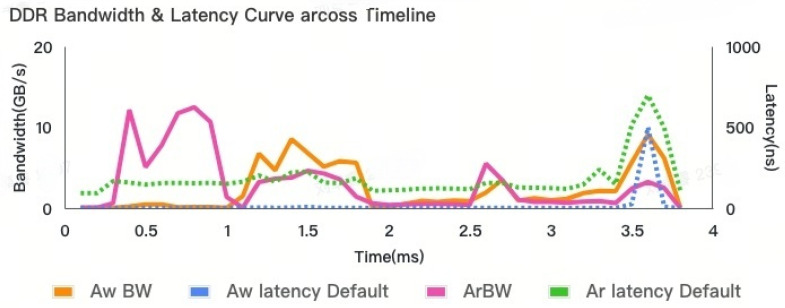
DDR bandwidth and latency curve across timeline.

In this section, we compare the rank-level read/write turnaround architecture with an adaptive control arbiter (ADCO) from a performance perspective. The work adopted in this article can be orthogonally and seamlessly embedded into the original read–write switching logic together with most of the read–write switching schemes available on the market. We compare the ADCO with the previous fixed rank-level arbitration method (FRAL). From the figure below, we can see that the bandwidth requirement and average latency results vary along the timeline. Compared with Figure 8, it follows the DDR dynamic latency curve. As the bandwidth requirement increases, the latency consumed also increases.

However, from this result without adaptive control, we observe that just before the benchmark is completed, the latency increases abnormally even when the total bandwidth requirement remains around 0.5 ms. The difference lies in the increased write bandwidth requirement. This is the use case behavior: at around 0.5 ms, the GPU engine reads a large amount of texture buffer to process the picture. At 3.5 ms, the GPU flushes the write buffer as the current frame is executed, and then the task for the next frame will begin.

**Figure 9:**
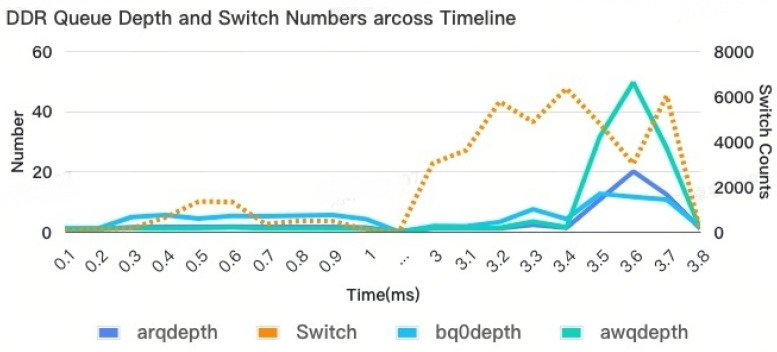
DDR BQ depth details.

This large bandwidth requirement further increases many read/write turnarounds. The details of the AQ and BQ depths in Figure 9 represent the internal loading details of the DDR controller. The depths of AQ and BQ can also offer insights into this trend. However, the increase in latency difference is not directly proportional to the increase in bandwidth. The relationship between the two is not consistent or in the same ratio.

The latency, especially the read latency, is increased by other factors rather than the bandwidth requirement.

The observed abnormal behavior can be ascribed to the alteration in data patterns. The conventional DDR control mechanism finds it difficult to handle this effectively. This is mainly due to the fact that the arbiter setting is fixed and thus cannot adapt to such variations.

**Figure 10:**
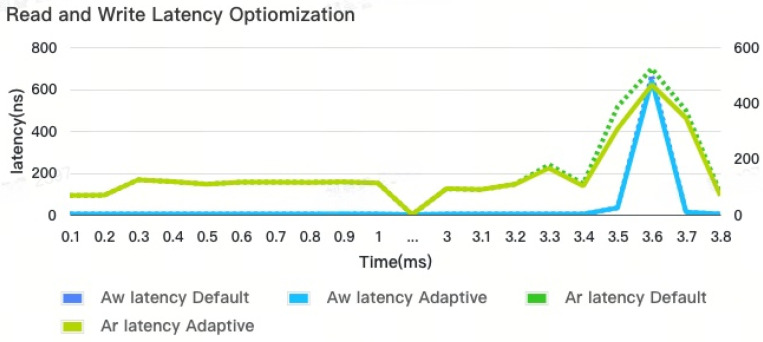
Read and write latency optimization.

Based on the provided read/write latency diagram in Figure 10, it can be observed that the adaptive control mechanism implemented in the DDR controller has significantly reduced the overall read latency by approximately 10 percent compared to a traditional DDR controller. At the sudden change pattern, the read latency is reduced from 697 ns to 620 ns. For write latency, it is reduced from 500.48 ns to 480 ns. In the normal part, the latency still maintains the same performance. This reduction in read latency indicates the effectiveness and improvement achieved by the adaptive control mechanism in optimizing the read latency performance.

### 4.3. Synthesis Results

Both the ADCO is implemented near the memory controller and does not introduce any changes to the DRAM chip or its interfaces. We evaluate the ADCO switch hardware complexity using Synopsys Design Compiler [15]. We implement ADCO in verilog and synthesize the emitted Verilog HDL design using Synopsys Design Compiler with a 5 nm process technology.

**Area Analysis.** ADCO consists of two main parts: a read/write mode arbiter and the dynamic switch logic, which consume 0.0067059 mm^2^ and 0.0012179 mm^2^ per memory channel at a 5 nm process technology, consuming an overall area overhead of 0.003% of a state-of-the-art Intel Xeon processor’s chip area [16] (which is implemented in an Intel 10 nm technology node) as well as the SOTA controller [17].

**Latency Analysis.** According to our RTL model, ADCO can be clocked at 1.6 GHz (0.625 ns). This latency is faster than the latency of regular memory controller operations as it is smaller than tRRD (e.g., 2.5 ns in DDR4 [18] and 5 ns in DDR5 [19]).

**Power Analysis.** The ADCO branch path as shown in Threshold Active and Idle Manager can be clocked at 25.6 MHz, which is directly from OSC. This is to save the power of the real-time monitoring overheads. As for power consumption variations under different workload conditions, the real-time monitoring overheads work at a much lower frequency reference clock compared with the core clock of 1.6 GHz. Power consumption is relatively low. The power consumption of the ADCO is 1.48 mW per memory channel at a 5 nm process technology, consuming an overall power overhead of 0.1% of a state-of-the-art SYNPS DDR controller read total power consumption.

## 5. Related Work

This section discusses other related works for Rank-Level Read/Write turnaround arbitration. Previous work on DRAM Rank-Level Read/Write turnaround like the DRAM cache work includes DRAM cache-based rank-level arbitration [20,21,22]. The proposed architecture, however, enables individual operations of each rank by introducing an MiB to every rank. Thereby, multiple ranks can work in parallel, leading to a creation of mRLP. The evaluations demonstrated that mRLP is effective for diverse configurations. Given the cost-sensitive DRAM market, a new DRAM architecture should be carefully designed.

DDR3 does not offer the rank-level switching timing, which could take advantage of the rank-level switching [23,24]. But as LPDDR5, which is the latest DRAM type, this type provides identical constraints to the rank-level timing. Thus, we further calculate the timing difference of it and timing control arbiter difference.

The HSSA [25,26,27] work proposes a highly parallel multi-level architecture for the open row command scheduler of a real-time SDRAM controller. The arbitration logic is distributed by utilizing the timing relationships of consecutive SDRAM commands. Experiments show that this architecture does not significantly change the performance of the scheduling algorithm. (1) There are indeed some common methodologies in the controller arbitering methodology, as they both apply the multi-stage architecture command scheduler. In the work of HSSA, in the CAS arbiter state, the SOTA memory controller masked out the other direction commands with the help of the timing constraints arbiters like tCCD, tBURST, tRTW, and tWTR. Thus, in this way, the multi-ranks arbitering is considered. This work also applied a more conservtive read/write switching policy, which leaves the timing constraints in a later stage. This work used the read/write turnaround mode to switch the read/write arbiter state with more vague timing constraints and calculation. This work further enhances the tRTW and tWTR to tRTW _s, trtw_d, twtr_s, twt_d and calculates the balance point with it. And it used the queue transaction status to imply this. Furthermore, the read–write switch is also impacted by the queue status, and the balancing point of the turnarounds is automatically switched. Regarding the ACT arbiter difference, to achieve the ACT arbiter, the round robin arbiter is not implied, but a very efficient ACT arbiter was implemented.

Overall, the reduction in read latency achieved by the adaptive control mechanism effectively demonstrates its role in enhancing read performance, thereby contributing to a more efficient and responsive DDR subsystem.

## 6. Conclusions

Our goal in this paper is to reduce the performance overheads of read/write turnarounds using the following: (1) a controller architecture decoupled from the scheduler and the protocol generator; (2) placing multiple arbitrators inside the address queue and the bank queue for different key commands, and (3) matching multiple algorithm engines with the timing mechanism of the protocol test to solve the adaptive demand problem. Going deep into the circuit level, the parallel method we adopt is more thorough than the industry’s solution, and it is a more in-depth multi-threaded form. Since each command generates read/write and activity/precharge commands at the same time, the activity/precharge commands are concurrently arbitrated, and the channel of key commands is another parallel structure that is different from the serial structure of the channel of read and write commands. The command arbitration is uniformly handed over to the protocol to ensure the high efficiency of DRAM. This is a more true multi-threaded approach, in which read/write commands are converted to precharge/activity commands, which are first arbitrated based on the priority factor of DDR utilization to ensure the highest DDR efficiency. When activation is achieved in the DRAM measurement to meet the timing parameters, the read/write commands continue to be arbitrated based on priority.

In our research, we propose an adaptive control architecture to enhance the performance efficiency of data movement in the memory subsystem. By addressing key factors and integrating parallelization and adaptive techniques, our aim is to optimize the memory subsystem for high-performance computing applications. This approach allows us to improve both the performance and efficiency of data movement within the memory subsystem and ultimately boost the overall system performance. Regarding dynamic threshold control strategies, we have also compared the performance benefits brought by this technology, which leads to average read latency savings ranging from approximately 10 nanoseconds to 150 nanoseconds.

In summary, our research centers on both the performance and power facets of the memory subsystem. Through comprehending the internal characteristics of DRAM that affect DDR utilization and conducting comprehensive throughput analysis, we guarantee the competitiveness of our proposition. Our results indicate that the Adaptive Performance Tuning DRAM controller effectively decreases read latency when compared with traditional DDR schedulers.

## Figures and Tables

**Table 1:** RD to WR turnaround penality.

SR/DR(6400)	Read	Write	Activite
READ	8/8+r2r_gap ^a2^	16/8+r2w_gap ^a1^	NA
WRITE	69/8+w2r_gap ^a1^	8/8+w2w_gap ^a2^	NA
ACTIVITE	NA	NA	6/0

^a1^ Generally, the r2r/w2r/r2w/r2r_gap is about 8 to 15 DRAM cycles across different timing speeds. ^a2^ t_r(w)2r(w)_gap = tPRST(tWPST) − 0.5tCK + CL(CWL) − CWL(CL) + tRPRE(tWPRE) + rd(wr)2rd(wr)_DQS_gap.

**Table 2:** Platform configuration.

Item	Configuration
ddrc commm num	8
ddrc comm period	18,760
ddrc comm timeout	1600
ddrc comm data width	32
ddrc data rate	4266
ddrc comm split byte	64
ddrc comm aw outstanding	256
ddrc comm ar outstanding	256
ddrc aw/wd/br/ar/rd delay	1
ddrc comm aw depth	2
ddrc comm wd depth	256
ddrc comm br depth	32
ddrc comm ar depth	2
ddrc comm rd depth	2048

## Data Availability

The original contributions presented in the study are included in the article, further inquiries can be directed to the corresponding author.
